# Piezoelectric Iron (II) Tungstate-Integrated Three-Dimensional-Printed Polycaprolactone Scaffolds for Ultrasound-Triggered Osteosarcoma Therapy and Ultrasound-Free Bone Repair

**DOI:** 10.34133/bmr.0420

**Published:** 2026-09-22

**Authors:** Jiongming You, Biaotong Huang, Yinsheng Wu, Xinxing Sun, Na Liang, Hao Wang, Yong Wang

**Affiliations:** ^1^ Department of Orthopedic, Wenzhou Hospital of Integrated Traditional Chinese and Western Medicine, Wenzhou 325000, Zhejiang, China.; ^2^ Wenzhou Institute, Shanghai University, Wenzhou 325000, Zhejiang, China.

## Abstract

The demand for effective therapeutics addressing osteosarcoma necessitates multifunctional solutions integrating both antineoplastic and osteogenic properties. This study presents a novel approach involving a 3-dimensional (3D)-printed polycaprolactone (PCL) scaffold integrated with piezoelectric iron (II) tungstate (FeWO_4_) nanorods (NRs), designed to concurrently combat osteosarcoma and foster bone regeneration. Through the amalgamation of FeWO_4_ NRs’ piezoelectric catalytic characteristics and PCL scaffold’s structural attributes, the composite scaffolds exhibit dual functionality: inducing oxidative stress in tumor cells via ultrasound-triggered piezo-sonoydnamic effects while facilitating stem cell attachment and proliferation without ultrasound. The physicochemical attributes and catalytic efficacy of the composite scaffolds are meticulously investigated. Both in vitro and in vivo tests confirm the strong ultrasound-triggered antitumor and ultrasound-free bone-forming effects of the PCL@FeWO_4_ composite scaffold. This study introduces a novel class of piezoelectric nanomaterial-functionalized 3D-printing scaffolds, presenting new avenues for ultrasound-triggered osteosarcoma therapy and ultrasound-free bone regeneration.

## Introduction

Osteosarcoma is the most common primary malignant bone tumor. Bone tumors that spread from the breast, lung, and kidney also seriously threaten patient survival [1–4]. Although surgery with chemotherapy enhances survival rates, fully eliminating tumors is difficult because of complex tissue structures, the aggressive nature of osteosarcoma, and chemotherapy side effects, which heighten the risk of recurrence and metastasis. Additionally, irregular bone defects post-tumor resection require innovative solutions. Therefore, it is vital to develop optimized artificial implants with potent antitumor performance and osteogenic capability for osteosarcoma, addressing both residual tumor cells and surgical defects.

Recently, an innovative approach has emerged through the integration of 3-dimensional (3D)-printed scaffolds with nanoscale biomaterials. This method synergistically combines the distinct advantages of both technologies to overcome the limitations associated with conventional bone scaffolds and therapeutic nanoagents [5–7]. 3D-printed scaffolds with customizable designs and strong macroporous structures aid stem cell growth and bone regeneration. Nanomaterials improve osteosarcoma treatments and modify the biological behavior of these constructs, offering varied therapeutic options and enhancing their functionality [8]. This combined approach offers an effective method for treating osteosarcoma and repairing bone defects. For instance, photothermal nanoagents, such as MXenes [9] and black phosphorus nanosheets [10], have been incorporated onto 3D-printing scaffolds to form dual-functional composite scaffolds, which can effectively eradicate residue tumor cells and accelerate the osteogenic process. However, the limited tissue penetration depth of near-infrared (NIR) light renders photonic hyperthermia ineffective in ablating osteosarcoma tumor cells satisfactorily. Therefore, it is crucially important to explore new therapeutic modalities with enhanced tissue penetration depth and desirable therapeutic efficacy to meet the growing demands for osteosarcoma-related therapeutic and regenerative applications.

Sonopiezoelectric therapy (SPT) is an emerging technology that utilizes piezoelectric nanomaterials [11]. These materials generate an electric field when exposed to ultrasound (US) irradiation. The US causes polarization in these nanomaterials, which separates charges and produces cytotoxic reactive oxygen species (ROS) via redox reactions. With its ability to penetrate tissues deeply, low-intensity US-triggered SPT shows promise as a noninvasive and repeatable treatment for deep-seated tumors. Additionally, it offers the advantage of selectively targeting cancer cells while minimizing damage to surrounding normal tissues, as US can be precisely focused on specific tissue targets of interest. Due to these advantages, SPT has garnered increasing attention for the treatment of deeply seated osteosarcoma [12]. However, the utilization of piezoelectric materials in the sequential therapy and bone regeneration of osteosarcoma remains largely unexplored. On one hand, the variety of piezoelectric nanomaterials is still limited, primarily focusing on BaTiO_3_ [13], ZnO [14], and polyvinylidene fluoride (PVDF) [15]. On the other hand, the single-modality approach of SPT may result in suboptimal killing effects against malignant tumor cells. Therefore, there is a pressing need to develop novel types of piezoelectric nanomaterials that can be integrated with 3D-printing scaffolds. This integration would enable both US-excited ROS generation and other lethal effects, enhancing the therapeutic efficacy of the nanoengineered composite scaffolds.

**Fig. 1.**
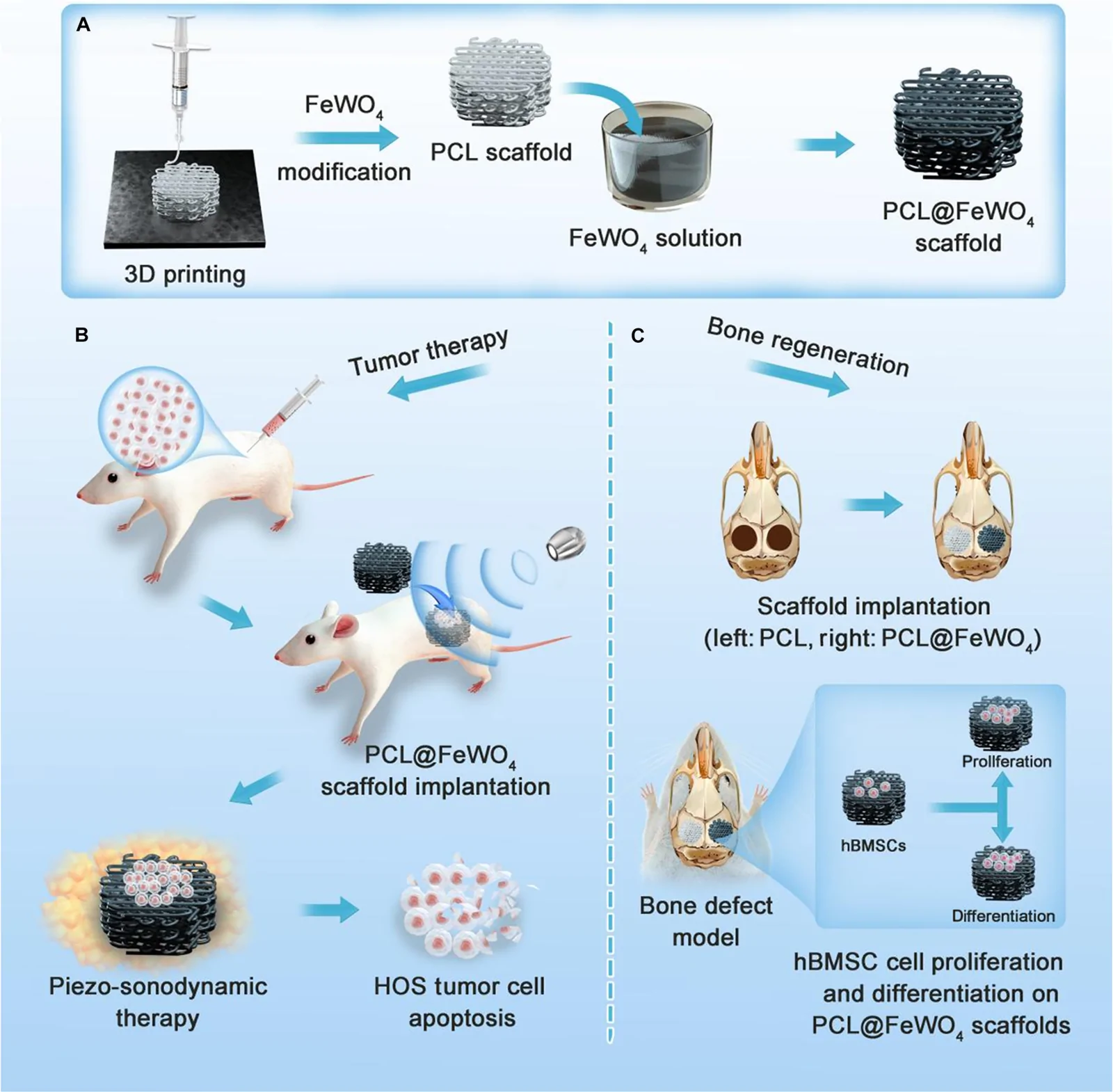
Schematic showing the preparation of a 3D-printed PCL@FeWO_4_ scaffold and its application in piezo-sonodynamic therapy for osteosarcoma and US-free bone repair in skull defects. (A) Preparation process of PCL@FeWO4 scaffolds. (B) Schematic illustration of PCL@FeWO4 scaffolds for osteosarcoma treatment. (C) Schematic illustration of PCL@FeWO4 scaffolds for the treatment of bone tissue regeneration.

Based on the aforementioned considerations, we hereby present the synthesis of iron (II) tungstate (FeWO_4_) nanorods (NRs), which serve as a novel class of piezoelectric nanoagents. These NRs are seamlessly integrated into a 3D-printed polycaprolactone (PCL) scaffold, enabling US-triggered SPT and catalytic therapy for osteosarcoma while independently supporting US-free accelerated bone regeneration (Fig. 1). The FeWO_4_ NRs possess dual functionalities. They can be stimulated by US irradiation to produce ROS, and simultaneously demonstrate catalytic activity by generating toxic hydroxyl radicals (•OH) upon encountering tumor-overexpressed hydrogen peroxide (H_2_O_2_). These ROS induce oxidative stress and exert cytotoxic effects, such as the disruption of membrane integrity, DNA damage, and protein denaturation in cells. Ultimately, this cascade of events results in efficient apoptosis of osteosarcoma cancer cells. The 3D-printed PCL scaffolds modified with FeWO_4_ NRs (PCL@FeWO_4_ scaffolds) have a staggered microstructure that supports the attachment and growth of human bone marrow mesenchymal stem cells (hBMSCs). Employing FeWO₄ NRs in a 3D-printed scaffold, compared with the widely studied BaTiO₃ [13], ZnO [14], and PVDF [15] systems, the PCL@FeWO₄ scaffold combines US-triggered sonopiezoelectric activity with catalytic •OH generation, achieving dual antitumor effects via a single nanoagent. Moreover, the scaffold’s osteogenic bioactivity does not require sustained US stimulation, enabling bone repair to proceed independently after initial tumor treatment.

In this study, the physicochemical properties and biocompatibility of the composite scaffolds were examined. The piezoelectric catalytic activity, antitumor effects, and bone-forming ability of the scaffolds were tested in vitro and in vivo. Results show that PCL@FeWO_4_ scaffolds can kill osteosarcoma cells and promote bone formation by hBMSCs. This study presents a piezoelectric catalytic 3D-printed scaffold for treating bone tumors and defects.

## Materials and Methods

### Materials

All scaffold preparation materials are from Shanghai Macklin Biochemical Co. Ltd. The rest came from Beyotime Biotech Inc.

### Synthesis of FeWO_4_

(NH₄)₂Fe(SO₄)₂·6H₂O (1.182 g) and Na₂WO₄·2H₂O (0.996 g) were separately dissolved in 5 ml of deionized water. Under continuous magnetic stirring, the Na₂WO₄ solution was added dropwise to the (NH₄)₂Fe(SO₄)₂ solution. Subsequently, 20 ml of deionized water was introduced, and stirring was maintained for approximately 30 min. The pH of the mixture was then adjusted to 9 using a 5 M NaOH solution, with stirring continued for an additional 40 min. The resulting suspension was transferred to a 50-ml reaction flask and heated in an oven at 160 °C for 24 h, after which it was allowed to cool to room temperature. The precipitate was collected by centrifugation (13,000 rpm, 10 min). The precipitate was washed 3 times alternately with ethanol and water, then freeze-dried and stored for later use.

### Preparation of PCL@FeWO_4_ scaffolds

A composite scaffold was prepared using the melt extrusion method. Firstly, the porous PCL scaffold was designed using computer-aided design (CAD) software and then printed using PanoSpace BiOne biological 3D printer (Beijing MinSu Intelligent Manufacturing Biotechnology Co. Ltd.). PCL pellets (molecular weight 80,000, Sigma, USA) were melted at 120 °C in a heating barrel. Finally, a 3D printing pattern was created using a metal nozzle with an inner diameter of 300 μm along the *z* axis at 0°|60°|120°. The cylindrical PCL scaffold model had a diameter of 5 mm and a height of 1.8 mm. The printing parameters used were as follows: the temperature of the heating barrel was 120 ± 15 °C; the air pressure during extrusion was 250 kPa; and the printing speed was 2 mm/s.

The printed PCL scaffold (5 mm diameter, 1.8 mm height) was cleaned ultrasonically for 10 min, soaked in FeWO_4_ solutions (4.0, 8.0, and 16.0 mg/ml) for 10 min, and then dried at room temperature. This soaking and drying process was repeated 3 times to produce the FeWO_4_ composite scaffold.

### Physicochemical characterization and ion release performance

Transmission electron microscopy (TEM), high-resolution transmission electron microscopy (HRTEM), and corresponding elemental mapping/electron-dispersive spectroscopy (EDS) were obtained using Tecnai G2 F20 (FEI, USA) at an accelerating voltage of 200 kV. Scanning electron microscopy (SEM) images were taken with Sigma 300 (Zeiss, Germany). The crystal structure and composition of FeWO_4_ were analyzed by x-ray diffraction (XRD; Rigaku Ultima IV, Japan) from 10° to 80°.

The chemical composition of FeWO_4_ was examined by x-ray photoelectron spectroscopy (XPS; Thermo Scientific K-Alpha, USA). Particle size distribution and surface charge of FeWO_4_ nanoparticles were measured using a nanosize and zeta potential analyzer (Zetasizer Pro, Malvern Panalytical, Netherlands).

Elemental mapping/EDS, surface morphology, mechanical, electromagnetic, and chemical properties of the composite scaffold were measured by field emission SEM (HITACHI SU8010, Japan) and atomic force microscopy (AFM; PFM, Dimension Icon, Bruker, Germany). Electron paramagnetic resonance (EPR) spectroscopy (EMXplus-6/1, Bruker, Germany) was used to detect O_2_^•−^, ^1^O_2_, and ·OH.

The compressive mechanical properties of the scaffold samples (PCL, PCL@FeWO_4_-L, PCL@FeWO_4_-M, and PCL@FeWO_4_-H) were evaluated using an Instron 5944 universal testing machine (Instron, USA). Cylindrical specimens were placed between the steel compression platens and subjected to a uniaxial compressive load at a constant crosshead speed at room temperature. The applied load and corresponding displacement were recorded, and the stress–strain curves were plotted. To minimize the effects of nonuniform contact at the onset of compression, the compressive modulus was calculated from the linear elastic region of the stress–strain curves. Specifically, the modulus value for each sample was determined by calculating the slope of the linear fit within the strain range of 5% to 15%.

The piezoelectric properties of the PCL@FeWO_4_-L, PCL@FeWO_4_-M, and PCL@FeWO_4_-H scaffolds were characterized using a ZJ-3A quasi-static piezoelectric coefficient measuring system. The scaffolds were cut into specimens of appropriate dimensions and subsequently subjected to electrical poling under a DC voltage of 2,000 V at room temperature for 1 h. The piezoelectric coefficient (*d*_33_) was measured immediately after the poling treatment.

PCL@FeWO₄-M scaffolds were weighed and individually immersed in 5 ml of phosphate-buffered saline (PBS) (pH 7.4) at 37 °C (*n* = 3). At designated time intervals (1, 2, 4, 6, 8, 12, 24, 36, 48, 72, 96, and 120 h), 2.5 ml of the release medium was withdrawn and immediately replenished with an equal volume of fresh PBS. The collected aliquots were immediately acidified with 2.5 ml of 4% dilute nitric acid. The Fe ion concentrations were determined using an inductively coupled plasma optical emission spectrometer (ICP-OES; PerkinElmer Avio 200). The cumulative release per unit mass was calculated, and the total Fe content within the scaffolds was quantified by the same ICP-OES (PerkinElmer Avio 200) following microwave digestion and appropriate dilution to derive the cumulative release percentage.

### DPBF and MB degradation evaluation by US activation

FeWO₄ (1 mg/ml) and 1,3-diphenylisobenzofuran (DPBF) (200 μM) were dispersed in 20 ml of ethanol and mixed thoroughly. The resulting dispersion was subjected to ultrasonication (3 MHz, 1.0 W/cm^2^, 50% duty cycle). At 5-min intervals, a 5-ml aliquot of the mixture was withdrawn and centrifuged, and the supernatant was collected. Ultraviolet–visible (UV–Vis) absorption spectra were recorded using a UV–Vis–NIR spectrophotometer (UV-3600 Plus, Shimadzu). The absorbance of DPBF at 411 nm was monitored over time, and under identical conditions, the absorbance of methylene blue (MB) at 664 nm was also measured. To prevent any photocatalytic degradation of DPBF and MB, all experiments were carried out in a dark room.

### Degradation of DPBF and MB by PCL@FeWO_4_ scaffolds

Different concentrations of PCL@FeWO_4_ scaffolds (0, 4.0, 8.0, and 16.0mg/ml) were placed in a 48-well plate. To each well, 0.5 ml of DPBF (200 μM) and H_2_O_2_ (10 mM) was added, and the absorbance of DPBF was measured every 5 min. Under the same conditions, the absorbance of MB at 664 nm was measured using the same method.

### TMB chromogenic assays

TMB chromogenic analysis was carried out in a 96-well plate at 25 °C. FeWO_4_ at concentrations of 0.02, 0.05, and 0.1 mg/ml was mixed with 10 mM TMB in the plate. Then, 0.1 M H_2_O_2_ was added, and absorbance at 652 nm was recorded every 30 s using a microplate reader (Infinite E Plex, TECAN). Under the same conditions, absorbance changes of 10 mM TMB were measured over time with FeWO_4_ at 0.05 mg/ml and different H_2_O_2_ concentrations (0.02, 0.1, and 0.5 M).

### In vitro cell viability and fluorescence imaging assay

SUNNCELL Co. Ltd. (Wuhan, China) provided the human osteosarcoma (HOS) osteosarcoma cells. Cells were grown in Dulbecco’s modified Eagle’s medium (DMEM) with 10% fetal bovine serum and 1% penicillin/streptomycin at 37 °C with 5% CO_2_. For the cytotoxicity assay, HOS cells (2 × 10^4^ cells per well) were gently seeded onto different scaffolds (total of 5 treatment groups: control, PCL, PCL@FeWO_4_-L, PCL@FeWO_4_-M, and PCL@FeWO_4_-H) in a 48-well plate, with each well containing 0.5 ml of culture medium. The cells were incubated for 24 and 48 h to allow them to adhere and proliferate on the scaffolds. Additionally, the 5 treatment groups were subjected to US (Chattanooga) irradiation at different power densities (0.5, 1.0, and 1.5 W/cm^2^) for 3 min, followed by standard Cell Counting Kit-8 (CCK-8) assays to validate the cell viability after each treatment.

For fluorescence imaging, HOS cells were seeded in a 48-well plate (2 × 10^4^ cells per well) and cultured for 24 h. Subsequently, the 5 types of scaffolds were gently placed into the wells, and US (1.5 W/cm^2^) was applied for 3 min. After removing the scaffolds, the cells were washed once with PBS and then stained with an appropriate volume of calcein AM/propidium iodide (PI) staining solution. The cells were then incubated at 37 °C in a cell culture incubator for 30 min to obtain live/dead cell staining results. Following the same treatment, the cell culture medium was removed, and an appropriate volume of 2,7-dichlorofluorescein diacetate (DCFH-DA) (sufficient to cover the cells) was added as a ROS fluorescent probe. The cells were then incubated at 37 °C in a cell culture incubator for 20 min to obtain ROS staining results. Fluorescence images were captured with a Nikon fluorescence microscope (ECLIPSE Ts2R-FL).

### Osteogenic differentiation of hBMSCs in vitro

hBMSCs from Cyagen Biosciences were grown in OriCell Complete medium at 37 °C, 5% CO_2_. hBMSCs (2 × 10^4^ cells per well) were gently seeded onto PCL or PCL@FeWO_4_ scaffolds (Ø 5 mm × 1.8 mm) in a 48-well plate, with each well containing 0.5 ml of culture medium, to allow the cells to adhere and proliferate on the scaffolds. After 1 and 7 d of hBMSC culture, the cells were fixed and stained with 4′,6-diamidino-2-phenylindole (DAPI) and Actin-Tracker Red (incubated at room temperature in the dark for 40 min). The fluorescent microscope was used to observe the blue fluorescence of the cell nuclei (DAPI) and the red fluorescence of the cell cytoskeleton (Actin-Tracker Red) to assess cell proliferation and differentiation on the different scaffold groups.

For osteogenic differentiation, after coculturing the cells with the scaffolds for 24 h, the culture medium was replaced with OriCell hBMSC osteogenic differentiation medium (HUXMX-90021) to induce differentiation.

To observe the changes in calcium salt deposition and alkaline phosphatase (ALP) activity during the osteogenic process, on day 21 of the induction, the cells were fixed and stained with Alizarin Red S (ARS) (incubated at room temperature for 20 min) to evaluate calcium salt deposition. The ALP activity in the cells was measured using an Alkaline Phosphatase Assay Kit.

To investigate the molecular mechanisms underlying osteogenic differentiation of hBMSCs, total RNA was isolated after 7 d of coculture with the scaffolds. Subsequently, gene expression levels of target genes were quantified using standard quantitative polymerase chain reaction (qPCR) techniques. The primer sequences employed in this study are listed in Table S1.

### In vivo sonodynamic anticancer evaluation

Four groups of female Balb/c nude mice (4 weeks old, *n* = 5 each) from Vital River (China) were used: control (PBS), PCL@FeWO_4_-M (8.0 mg/kg), US (3 MHz; 1.5 W/cm^2^; 3 min; 50% duty cycle), and PCL@FeWO_4_-M + US. HOS cells (2 × 10^6^ in PBS) were injected subcutaneously. After 7 d, when tumors reached about 150 mm^3^, scaffolds (Ø 5.0 mm × 1.5 mm) were implanted under tumors under anesthesia. Tumor volume was measured every 2 d for 14 d with digital calipers and calculated as *V*_t_ = *lw*^2^/2 − *V* (*l* = length, *w* = width, *V* = scaffold volume). Relative tumor volume *V V*_r_ = *V*_t_/*V*_0_ (*V*_0_ = volume on day 0). On day 14, tumors were excised, weighed, fixed in 4% formalin, sectioned, and stained with hematoxylin and eosin (H&E), terminal deoxynucleotidyl transferase–mediated deoxyuridine triphosphate nick end labeling (TUNEL), and Ki-67 for pathology. Major organs were also collected, fixed, and H&E stained to check toxicity.

### In vivo osteogenesis investigation of PCL@FeWO_4_ scaffolds

#### Construction of calvarias defect models

All male Sprague–Dawley rats (8 weeks old) were obtained from Vital River (China). Animal experiments were approved by the Ethics Committee of Science and Technology of Wenzhou Institute of Shanghai University (ECWISHU 2023-005). Twelve male Sprague–Dawley rats (8 weeks old, 300 to 350 g) were anesthetized with pentobarbital. A 1.5- to 2.0-cm scalp incision was made. Two 5-mm-diameter full-thickness defects were created on each side of the skull. PCL scaffold was implanted on the left defects, and PCL@FeWO_4_-M scaffold (5 mm × 1.8 mm) was implanted on the right. Soft tissues were sutured with 4-0 silk, and rats received antibiotics post-surgery.

#### Micro-CT evaluation and corresponding histomorphometric analyses

At 4, 8, and 12 weeks post-surgery, rats were euthanized. Skulls were fixed in 4% paraformaldehyde and scanned by micro-computed tomography (CT). 3D images were reconstructed. Bone volume to tissue volume ratio (BV/TV), bone mineral density (BMD), and bone mineral content (BMC) were measured using CTAn software. Fixed bones were embedded, sectioned, and stained with H&E and Masson’s trichrome.

### Statistical analysis

The data are presented in the form of the mean value along with its corresponding standard deviation (SD). All experiments were performed with a minimum of 3 independent replicates (*n* ≥ 3). Statistical analysis for 3 or more subgroups was conducted using one-way analysis of variance (ANOVA), followed by Tukey’s multiple comparison test. For comparisons between 2 groups, a paired Student’s *t* test was applied. Significance levels were denoted as follows: *P < 0.05; **P < 0.01; ***P < 0.001; ns, not significant (*P* > 0.05). GraphPad Prism 10 software was employed for both graphing and analysis.

## Results and Discussion

### Fabrication and characterization of FeWO_4_ NRs

**Fig. 2.**
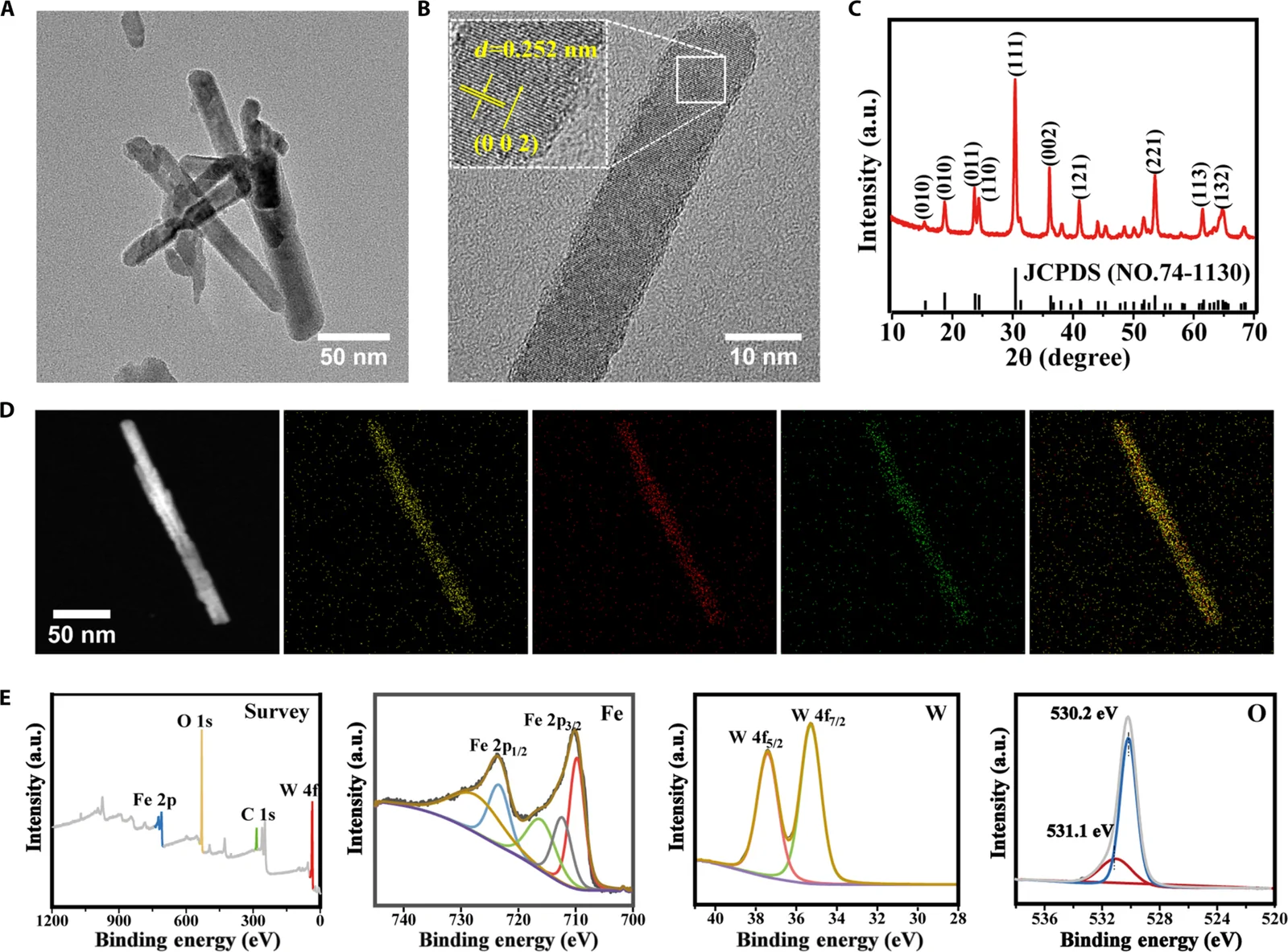
Characterization of FeWO_4_ NRs. (A) TEM image, (B) HRTEM image, and (C) XRD pattern of FeWO_4_ NRs. (D) STEM image and the corresponding elemental mapping images. (E) Survey XPS spectrum of FeWO_4_ NRs and high-resolution spectra of Fe 2p, W 4f, and O 1s.

To fabricate composite 3D-printing scaffolds, FeWO_4_ NRs were initially synthesized via a hydrothermal method at 160 °C (Fig. S1) [16,17]. The morphology of the FeWO_4_ nanostructures was confirmed to be 1D through TEM analysis (Fig. 2A). The phase purity and crystal structure of the FeWO_4_ NRs were further characterized using XRD (Fig. 2C). Moreover, a high-resolution TEM image (Fig. 2B) revealed the highly crystalline nature of the FeWO_4_ NRs, displaying distinct lattice fringes with a plane spacing of 0.252 nm, corresponding to the (002) atomic plane of γ-FeWO_4_.

The chemical composition of FeWO_4_ was assessed using EDS elemental mapping and XPS. Elemental mapping revealed a uniform distribution of Fe, W, and O within FeWO_4_ NRs (Fig. 2D), while EDS analysis confirmed the coexistence of Fe, W, and O signals (Fig. 2E), validating the successful synthesis of FeWO_4_. XPS spectra further substantiated the presence of Fe, W, and O elements across a broad energy range (Fig. 2E) [2,18–20]. The uniform size distribution of FeWO_4_ NRs was also validated by dynamic light scattering results (Fig. S2).

### Fabrication and characterization of PCL@FeWO_4_ scaffolds

**Fig. 3.**
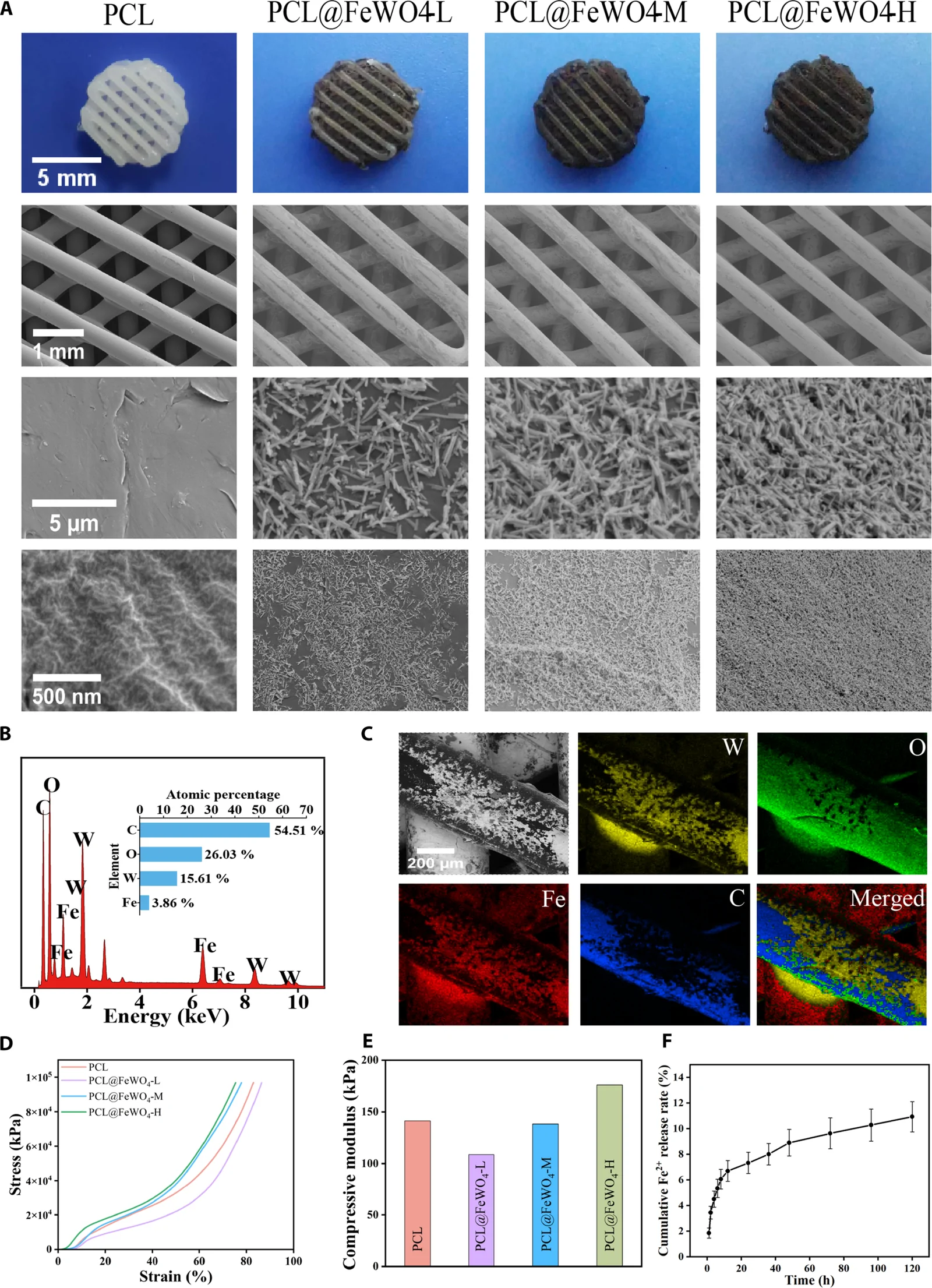
Characterizations of PCL@FeWO_4_ scaffolds. (A) Digital photographs and SEM images of PCL@FeWO_4_ scaffolds with 3D geometrical structure. From left to right, pure PCL scaffold and PCL@FeWO_4_ scaffolds immersed with FeWO_4_ dispersions at different concentrations (4.0, 8.0, and 16.0 mg/ml, corresponding to PCL@FeWO_4_-L, PCL@FeWO_4_-M, and PCL@FeWO_4_-H, respectively). From up to down, images of PCL@FeWO_4_ scaffolds at different magnifications (scale bar, 5 mm, 1 mm, 5 μm, and 500 nm). (B) EDS results and atomic percentage of PCL@FeWO_4_ scaffolds. (C) Corresponding elemental mappings of Fe, W, C, and O. (D) Compressive stress–strain curves of the pure PCL and PCL@FeWO_4_ composite scaffolds with varying FeWO_4_ concentrations. (E) Compressive modulus of the pure PCL and PCL@FeWO_4_ composite scaffolds. The modulus values were calculated from the linear elastic region within the strain range of 5% to 15%. (F) Cumulative release profile of Fe ions from the PCL@FeWO_4_-M composite scaffold incubated in PBS over time.

Subsequently, we utilized CAD software to create a porous 3D-printed PCL scaffold, which was then impregnated with ethanol solutions of synthesized FeWO_4_ NRs to form the PCL@FeWO_4_ scaffold (Fig. S3). By adjusting the concentration of FeWO_4_ NRs, we obtained PCL scaffolds with varying FeWO_4_ concentrations, denoted as PCL@FeWO_4_-L (4.0 mg/ml), PCL@FeWO_4_-M (8.0 mg/ml), and PCL@FeWO_4_-H (16.0 mg/ml). Microscopic analysis was performed to closely examine the surface morphology of the PCL@FeWO_4_ scaffold. SEM images revealed a 3D structure on the scaffold surface, characterized by multiple layers stacked in an interlocking and staggered manner in different orientations, forming a uniform and interconnected microstructure that enhances the mechanical stability of the scaffold (Fig. 3A). SEM images of PCL@FeWO₄ obtained after immersing the scaffold in ethanol solutions of FeWO₄ at different concentrations show that rod-shaped FeWO₄ particles are uniformly distributed across the scaffold surface, indicating that this loading method enables uniform nanoparticle loading. This straightforward surface modification strategy ensured the incorporation of FeWO_4_ NRs into the scaffold without significantly affecting its original structure. The highly ordered large pores and scattered surface morphology of the PCL@FeWO_4_ scaffold facilitate osteogenesis and bone integration, enhancing the interlocking between the scaffold and the surrounding natural bone [21,22]. When FeWO_4_ NR concentration increases, the scaffold color changes from white to black, with more FeWO_4_ NRs on the surface. EDS analysis confirmed the elements present on the scaffold surface (Fig. 3B). Elemental mapping also demonstrated the uniform distribution of Fe, W, C, and O elements, indicating the successful binding of the PCL scaffold with FeWO_4_ (Fig. 3C). The adhesion and uniform spreading of FeWO_4_ NRs on the surface of the PCL scaffold may confer intriguing catalytic performance and osteogenic ability to the composite scaffolds.

Compression tests revealed that the PCL@FeWO₄-H composite exhibited the highest mechanical performance, while the PCL@FeWO₄-L group showed a slightly lower compressive modulus compared to the pure PCL scaffold (Fig. 3D and E). This reduction at low doping levels is likely attributed to the agglomeration of FeWO₄ nanoparticles acting as stress concentrators and weak interfacial bonding, which hinders effective load transfer within the matrix. It is worth noting that the inherent mechanical strength of the bare PCL scaffold was already sufficient to meet the structural requirements for bone defect repair applications [23]. Therefore, considering both the adequate mechanical support and the comprehensive biological performance (combined with the subsequent in vitro cellular experimental results), the intermediate PCL@FeWO₄-M group was selected as the optimal candidate for the subsequent in vivo experiments. The in vitro cumulative Fe release profile of PCL@FeWO_4_-M showed a sustained release behavior without significant burst release, suggesting that the FeWO_4_ NRs remained relatively stable within the scaffold. This sustained release might provide a continuous supply of iron ions throughout the treatment process, which is beneficial for maintaining long-term therapeutic efficacy (Fig. 3F).

### Piezo-sonodynamic and catalytic performance of FeWO_4_ NRs and PCL@FeWO_4_ scaffolds

**Fig. 4.**
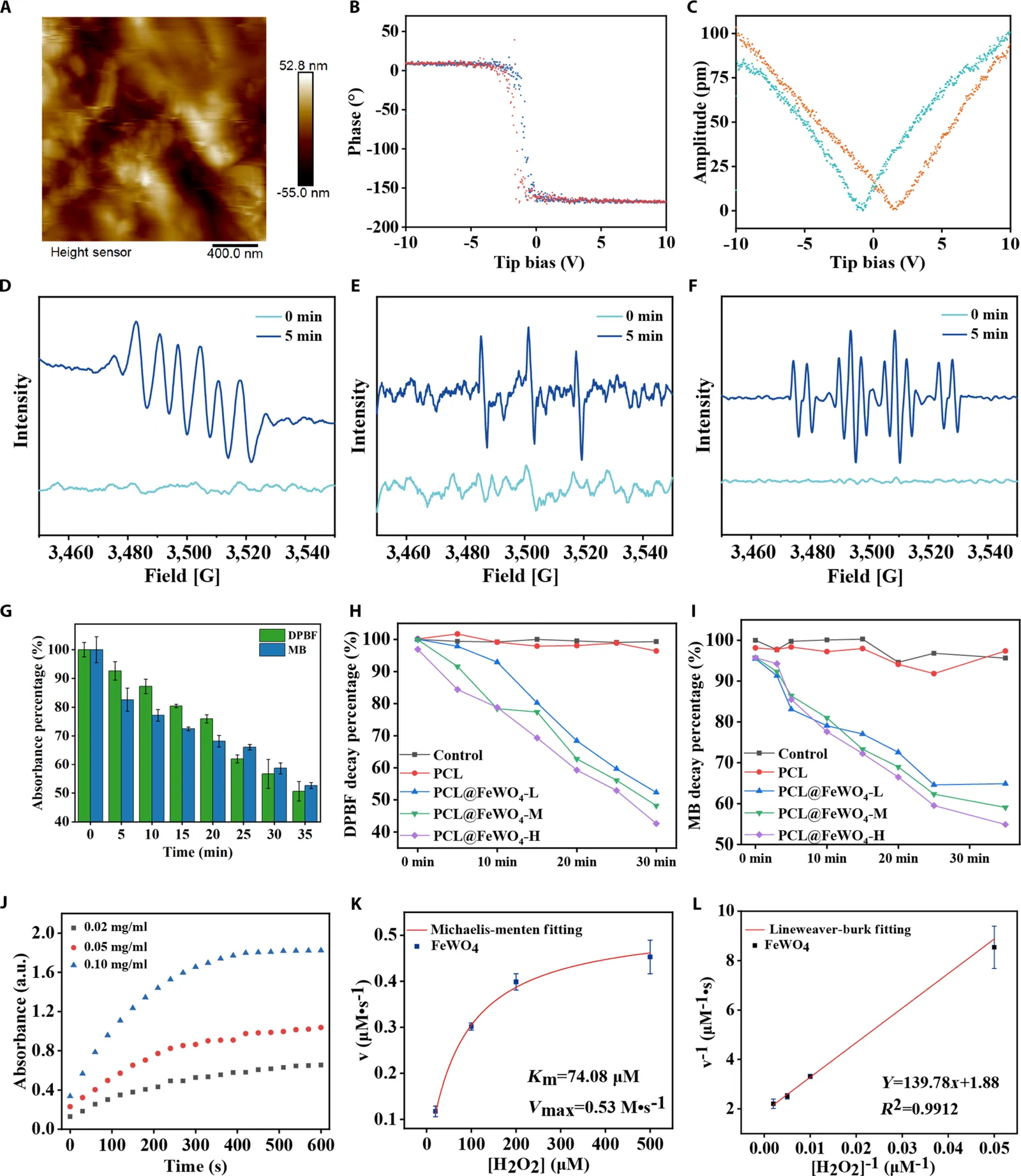
Piezoelectric and catalytic performance of PCL@FeWO_4_ scaffolds. (A) Piezoelectric force microscopy (PFM) image of the as-prepared PCL@FeWO_4_. PFM characterization of PCL@FeWO_4_ scaffolds showing (B) the phase and (C) displacement response. Electron spin resonance (ESR) spectra detecting ROS generation by FeWO_4_ NRs under US irradiation: (D) O_2_^•−^ , (E) ^1^O_2_, and (F) •OH signals. (G) Absorbance change of DPBF (20 μM) at 410 nm and MB (20 μM) at 665 nm at different time under US irradiation (1 W/cm^2^) when co-incubated with 1 mg/ml FeWO_4_. Degradation spectra of (H) DPBF and (I) MB by pure PCL, PCL@FeWO_4_-L, PCL@FeWO_4_-M, and PCL@FeWO_4_-H scaffolds incubated with H_2_O_2_ (1 mM). (J) Time-dependent absorbance of the chromogenic TMB when incubated with H_2_O_2_ in the presence of FeWO_4_ at varied concentrations (0.02, 0.05, and 0.1 mg/ml). (K) Michaelis–Menten fitting curve and (L) corresponding calculated Lineweaver–Burk fitting curve for the kinetic measurement of the TMB chromogenic profile for PCL@FeWO_4_ scaffolds incubated with H_2_O_2_ at varied concentrations.

The piezoelectric property of the PCL@FeWO_4_ scaffolds was assessed using piezoresponse force microscopy (PFM). This technique involves high-resolution scanning of the scaffold surface with a scanning probe, providing detailed insights into the morphology, microstructure, and surface characteristics of the PCL@FeWO_4_ scaffold (Fig. 4A and Fig. S4). Analysis of the piezoelectric response intensity and direction confirmed the piezoelectric features of the scaffolds, as evidenced by the PFM phase and amplitude curves (Fig. 4B and C), respectively. The phase image of the PCL@FeWO_4_ scaffold exhibited clear phase contrast, with the image contrast being opposite to the amplitude signal. Upon application of an alternating voltage to the PCL@FeWO_4_ scaffold, a hysteresis amplitude–voltage curve resembling a butterfly loop, characteristic of ferroelectric materials, was observed [24]. The average phase contrast, as depicted in the phase image, was approximately 262°, further confirming the piezoelectric nature of the PCL@FeWO_4_ scaffold [25]. The piezoelectric coefficient (*d*_33_) is a key parameter that quantifies the charge generated in response to mechanical deformation under an applied electric field [26]. As the FeWO_4_ content increased, the *d*_33_ values exhibited a gradual increase, demonstrating that the incorporation of FeWO_4_ nanofillers effectively enhanced the piezoelectric response of the PCL-based composite scaffolds (Fig. S5).

To assess the capacity of FeWO_4_ NRs to generate ROS under US stimulation, electron spin resonance (ESR) was utilized for quantitative measurements [27–33]. The FeWO_4_ solution underwent US stimulation, and the production of superoxide anion (O_2_^•−^), singlet oxygen (^1^O_2_), and hydroxyl radicals (•OH) was monitored at 0 and 5 min using 5,5-dimethyl-1-pyrroline N-oxide (DMPO) and 2,2,6,6-tetramethyl-4-piperidine (TEMP) as signal probes. Compared to the non-US condition, distinct peaks with robust intensity signals of O_2_^•−^, ^1^O_2_, and •OH were evident after 5 min of US exposure (Fig. 4D to F), indicating a notable piezoelectric effect of FeWO_4_ NRs under US excitation, highlighting their significant sonochemical performance.

Furthermore, the degradation of DPBF [34] and MB [35] solutions by FeWO_4_ NRs under US excitation was assessed to investigate the sonochemical performance of FeWO_4_ NRs (Fig. 4G and Fig. S6A and B). The characteristic absorbance of DPBF at 411 nm and MB at 664 nm exhibited a gradual decrease with increasing US excitation time, providing evidence that the production of ROS is associated with the US-triggered piezoelectric response of FeWO_4_ NRs. The ROS generation capability of the PCL@FeWO_4_ scaffold under US irradiation was also evaluated using DPBF and MB. Consistently, a decreasing trend in maximum absorbance values was observed across the different concentrations of PCL@FeWO_4_ scaffolds. Under identical US irradiation conditions, the DPBF degradation percentages were determined to be 47.74%, 51.75%, and 54.30% within 30 min for the PCL@FeWO_4_-L, PCL@FeWO_4_-M, and PCL@FeWO_4_-H scaffolds, respectively (Fig. 4H). In contrast, the control and PCL scaffold groups exhibited only slight absorption decreases. This suggests that higher concentrations of FeWO_4_ NRs in the PCL@FeWO_4_ scaffold lead to more significant reductions in fluorescence intensity of DPBF at 411 nm. Similarly, a comparable trend in fluorescence intensity changes at 664 nm for MB was observed in the 3 groups of PCL@FeWO_4_ scaffolds compared to the control and PCL scaffold groups (Fig. 4I). These findings indicate that PCL@FeWO_4_ scaffolds with higher concentrations of FeWO_4_ NRs possess superior ROS generation capabilities compared to those with lower concentrations.

The catalytic performance of FeWO_4_ NRs as a Fenton-like nanocatalyst was assessed through a colorimetric experiment employing tetramethylbenzidine (TMB) as the indicator. When TMB is exposed to ROS (such as hydrogen peroxide or superoxide radicals), it undergoes an oxidation reaction, which can be quantified by observing changes in absorbance of the TMB solution at 652 nm [36]. FeWO_4_ samples at different concentrations (0.02, 0.05, and 0.1 mg/ml) were incubated with 0.1 M H_2_O_2_ solutions, and the changes in characteristic absorbance of TMB over time were monitored. It was observed that higher concentrations of FeWO_4_ NRs exhibited an accelerating effect on ROS generation, indicating the efficient production of •OH by FeWO_4_ NRs (Fig. 4J). Furthermore, the results indicated that increasing H_2_O_2_ concentration enhanced the oxidation of TMB at a fixed FeWO_4_ NR concentration (0.05 mg/ml) (Fig. S7). Kinetic analysis using Michaelis–Menten and Lineweaver–Burk methods revealed a maximum TMB oxidation rate (*V*_max_) of 0.53 M·s^−1^ and a Michaelis constant (*K*_m_) of 74.08 μM (Fig. 4K and L). These findings demonstrate the strong Fenton-like catalytic activity of the FeWO_4_ NRs.

### In vitro therapeutic effects of PCL@FeWO_4_ scaffolds

**Fig. 5.**
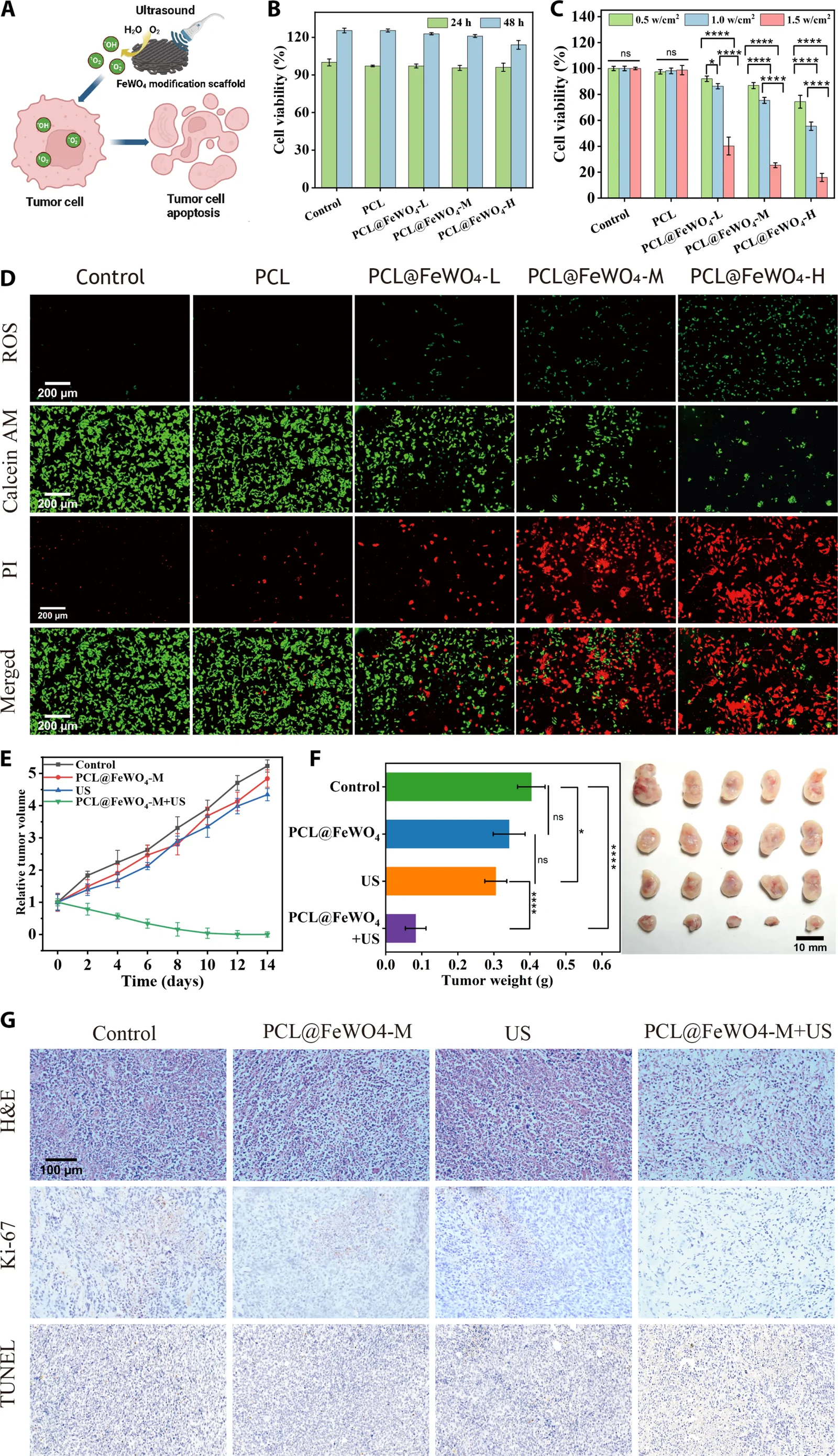
Piezo-sonodynamic and chemodynamic therapeutic effects of PCL@FeWO_4_ scaffolds against osteosarcoma. (A) Schematic illustration of osteosarcoma treatment enabled by US excitation of PCL@FeWO_4_ scaffolds. (B) Relative cell viability profiles of HOS cells co-incubated with different samples for 24 and 48 h, respectively. (C) Relative cell viability profiles of HOS cells treated with different samples upon US irradiation (3 min) at different power densities. (D) DCFH-DA staining and live-dead staining fluorescent images of HOS cells treated with different samples upon US irradiation. (E) Time-dependent tumor-growth curves of experimental mice after different treatments (control, PCL@FeWO_4_, US, and PCL@FeWO_4_ + US). (F) Average weights and photographs of tumors dissected from representative mice post-treatments. Scale bar, 10 mm. (G) Pathological assays of mice in each group via H&E, Ki-67, and TUNEL staining. Scale bar, 100 μm. Significance levels were denoted as follows: *P < 0.05; **P < 0.01; ***P < 0.001; ns, not significant (*P* > 0.05).

Motivated by the piezo-sonodynamic and catalytic capabilities of FeWO_4_ NRs demonstrated previously, in vitro cell experiments were conducted to assess their effectiveness in inducing lethal damage to osteosarcoma cells (Fig. 5A). The initial focus was on evaluating the cytotoxicity of PCL@FeWO_4_ scaffolds. HOS cells were divided into 5 groups (control, PCL, PCL@FeWO_4_-L, PCL@FeWO_4_-M, and PCL@FeWO_4_-H) and cultured for 24 or 48 h to observe HOS cell proliferation. After 24 h of cell culture, the cell viability in each group approached 100%. Comparatively, the number of HOS cells notably increased after 48 h of culture, indicating that PCL@FeWO_4_ scaffolds exhibit favorable cytocompatibility and do not adversely affect cell growth and proliferation (Fig. 5B). As shown in Fig. 5C, US irradiation caused little damage to HOS cells whether PCL scaffolds were present or not. However, in groups treated with PCL@FeWO_4_ scaffolds, cell viability significantly decreased upon application of US, with a notable reduction correlating with increased FeWO_4_ NR concentration on the scaffolds at the same power density of US. For instance, the remaining cell viabilities of the PCL@FeWO_4_-L, PCL@FeWO_4_-M, and PCL@FeWO_4_-H groups were 40.20%, 25.37%, and 15.93%, respectively, at a power density of 1.5 W/cm^2^, demonstrating dose-dependent killing effects of SPT. Furthermore, the cell mortality rate increased proportionally with the power densities of US irradiation. These findings underscore the high piezo-sonodynamic efficacy of PCL@FeWO_4_ scaffolds in eradicating osteosarcoma cells.

Furthermore, the intracellular ROS generation induced by the piezo-sonodynamic effects of PCL@FeWO_4_ scaffolds under US excitation was examined using the probe DCFH-DA. As anticipated, HOS cells treated with PCL@FeWO_4_ scaffolds exhibited the most intense green fluorescence of 2,7-dichlorofluorescein under US irradiation compared to other groups (Fig. 5D), indicating abundant and toxic ROS generation. Moreover, the overall fluorescence intensity of intracellular ROS staining increased with the concentration of FeWO_4_ NRs on the PCL@FeWO_4_ scaffold (Fig. S8A).

Additionally, calcein AM and PI staining were utilized to observe the distribution of live and dead cells in different treatment groups. Fluorescent staining images revealed a distinct contrast in green or red fluorescence emitted by live and dead cells in various treatment groups after US treatment (Fig. 5D). The PCL@FeWO_4_-H + US group exhibited the highest proportion of red fluorescence in treated cells, indicating a positive correlation between the killing effect of composite scaffolds on HOS cells and the concentration of doped FeWO_4_ NRs. Conversely, in the control (US only) group and the pure PCL scaffolds + US group, the proportions of dead cells were only 4% and 24%, respectively. Notably, the PCL@FeWO_4_-H + US group showed a significantly increased proportion of dead cells, reaching 86% (Fig. S8B). These findings underscore the promising therapeutic effect of PCL@FeWO_4_ scaffolds, laying a foundation for their in vivo antitumor applications.

### In vivo tumor inhibition effect of PCL@FeWO_4_ scaffolds

Next, we evaluated the in vivo tumor inhibition effects of PCL@FeWO_4_-M scaffolds using a subcutaneous tumor model established in female Balb/c nude mice. It is worth mentioning that PCL@FeWO_4_-M composite scaffolds were selected due to the enhanced cell proliferation of hBMSCs on their surface compared to other composite scaffolds, as will be demonstrated in the next section. HOS cells were subcutaneously implanted into mice and permitted to establish tumors over a 7-d period, after which the animals were randomly assigned to 4 experimental groups, and subsequent measurements of body weight and tumor volume were conducted at 2-d intervals throughout the treatment course. Compared to the control group, the body weight of all mice showed little change (Fig. S9), suggesting that PCL@FeWO_4_-M scaffolds had minimal side effects. Analysis of the relative tumor volume curve revealed that tumors in the PCL@FeWO_4_-M scaffolds + US group gradually disappeared and showed no recurrence during the 14-d observation period (Fig. 5E), indicating the most significant anticancer effect. In contrast, rapid tumor growth was observed in the control, PCL@FeWO_4_-M scaffolds, and US groups over the same period.

After the treatments, the tumor tissues of experimental mice were excised and processed for histological staining. Additionally, the major organs of the nude mice were collected and sectioned for pathological analysis. The average weight and visual images of tumor tissues revealed that PCL@FeWO_4_-M plus US irradiation resulted in the smallest tumor size (Fig. 5F). Hematoxylin and eosin (H&E) staining of major organ sections (heart, liver, spleen, lung, and kidney) indicated no significant pathological abnormalities across all experimental groups (Fig. S10). These findings collectively indicate that the constructed composite scaffold exhibits high biocompatibility, thereby demonstrating the satisfactory effectiveness of PCL@FeWO_4_-M scaffolds for local piezo-sonodynamic therapy of osteosarcoma.

The collected tumor tissue sections underwent pathological analysis, including immunofluorescent Ki-67 staining, H&E staining, and TUNEL staining (Fig. 5G). Microscopic images revealed a higher proportion of Ki-67-negative cells in samples treated with PCL@FeWO_4_-M upon US irradiation, indicating efficient suppression of cell proliferation activity by the treatment. Additionally, elevated TUNEL expression in the piezo-sonodynamic group signaled significant apoptosis in osteosarcoma cells following treatment. Likewise, tumor tissues from the PCL@FeWO_4_-M + US group showed a reduced number of tumor cell nuclei in H&E staining images relative to the other 3 treatment groups. These results suggest that subcutaneously implanted PCL@FeWO_4_-M scaffolds can effectively eliminate tumor tissue under US excitation with high biocompatibility. It should be noted, however, that the subcutaneous xenograft model employed herein, while allowing convenient and reproducible monitoring of tumor volume, does not fully recapitulate the complex bone microenvironment characteristic of orthotopic osteosarcoma. Consequently, the antitumor efficacy observed in this model should be interpreted within this context, and further validation using orthotopic osteosarcoma models is warranted to better assess the therapeutic potential of the PCL@FeWO₄ scaffold under clinically relevant conditions. Therefore, PCL scaffolds integrated with FeWO₄ NRs hold promise as an ideal piezo-sonodynamic platform against osteosarcoma while recognizing that orthotopic evaluation represents an essential next step toward clinical translation.

### In vitro osteogenesis assessment of PCL@FeWO_4_ scaffolds

**Fig. 6.**
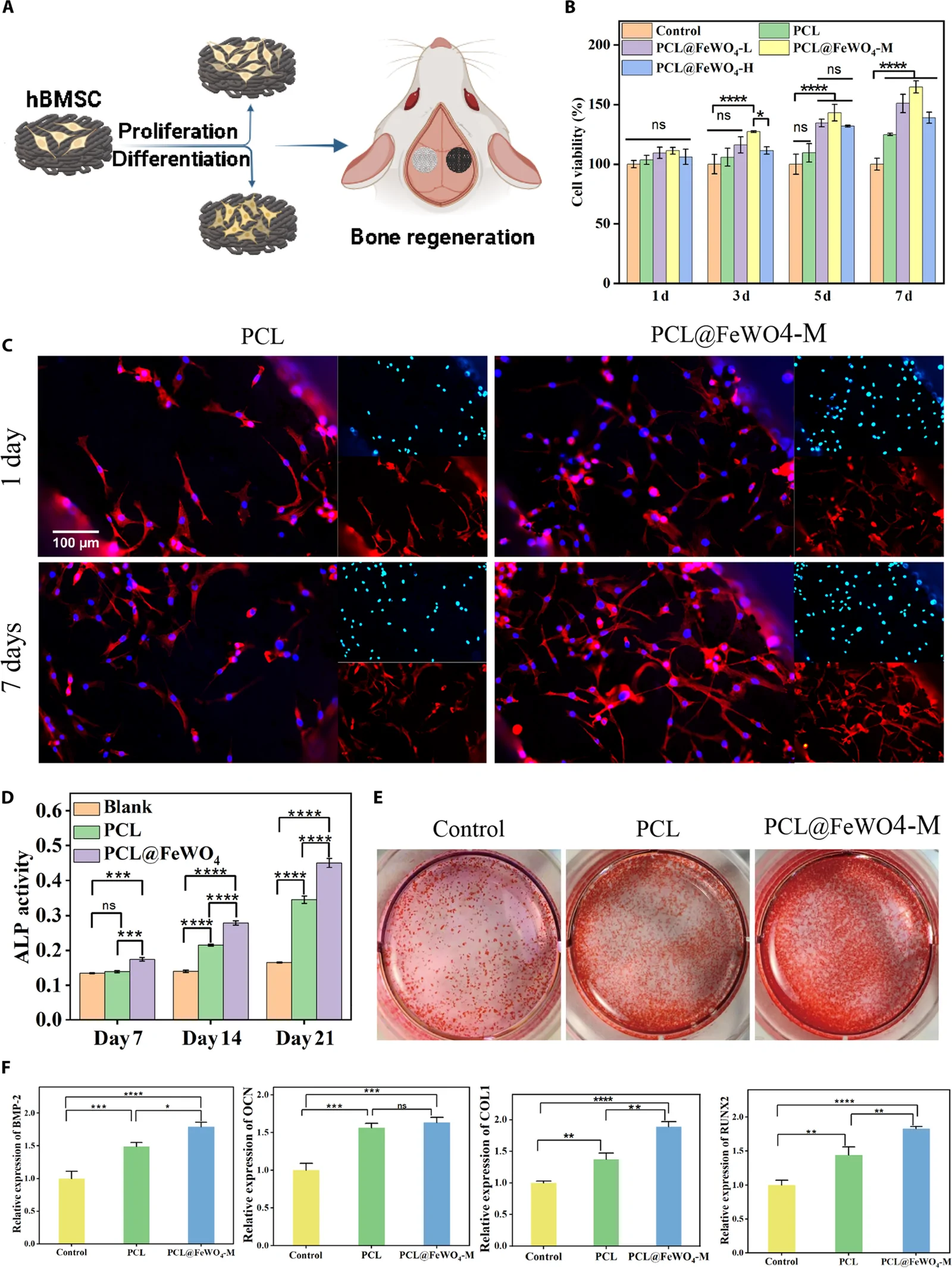
In vitro evaluations of osteogenic bioactivities of PCL@FeWO_4_ scaffolds. (A) Schematic illustration of the co-incubation of hBMSCs with PCL@FeWO_4_ scaffolds. (B) Relative cell viability profiles of hBMSCs treated with different samples for 1, 3, 5, and 7 d. (C) Confocal images of hBMSCs stained with DAPI (cell nuclei, blue fluorescence) and rhodamine phalloidin (cytoskeleton, red fluorescence) on pure PCL and composite PCL@FeWO_4_ scaffolds for 1 and 7 d. (D) Relative ALP activities of hBMSCs incubated with PCL@FeWO_4_-M scaffolds under osteoinduction conditions for 7, 14, and 21 d. (E) Alizarin Red S staining images of hBMSCs incubated with different samples after 21 d. (F) Osteogenic gene expression (COL1, OCN, BMP-2, and RUNX2) of hBMSCs in control, PCL, and PCL@FeWO_4_-M scaffolds at day 7. Significance levels were denoted as follows: *P < 0.05; **P < 0.01; ***P < 0.001; ns, not significant (*P* > 0.05).

The osteogenic potential of PCL@FeWO_4_ scaffolds was systematically assessed next. Initially, the composite scaffolds were cocultured with hBMSCs to observe cell proliferation and differentiation on the scaffolds (Fig. 6A). Notably, CCK-8 results indicated that PCL@FeWO_4_ scaffolds promote hBMSC cell proliferation compared to bare PCL scaffolds (Fig. 6B). Following 1 and 7 d of incubation, the cells were fixed and stained with DAPI and Actin-Tracker Red-Rhodamine to visualize cell nuclei (blue fluorescence) and cytoskeleton (red fluorescence) under a fluorescence microscope. The fluorescence staining images demonstrated that both PCL and PCL@FeWO_4_-M groups support the growth of hBMSCs, while the cell quantity on PCL@FeWO_4_-M scaffolds exhibited a significant increase, displaying elongated cell morphology, enhanced filamentous pseudopodia extension, and stronger adhesion to the scaffold (Fig. 6C and Fig. S11). These results indicate that PCL@FeWO_4_ scaffolds enhance hBMSC proliferation, potentially supporting osteogenic differentiation. ALP serves as a marker enzyme for mature osteoblasts, reflecting their quantity and thus indicating the state of bone formation. To induce osteogenic differentiation, hBMSCs were cocultured with the scaffolds for 24 h, followed by the application of specialized induction medium. After 7, 14, and 21 d, cells were lysed to extract ALP for quantitative analysis. As depicted in Fig. 6D, ALP activity in the PCL@FeWO_4_-M scaffold group significantly surpassed that of both the control and PCL scaffold groups at each time point. Mineralized nodules represent another hallmark of mature osteoblast differentiation. To visualize extracellular matrix mineralization during bone formation, on the 21st day of induction differentiation, cells were fixed and stained with ARS to assess calcium salt deposition. The relative intensity of ARS staining at days 7, 14, and 21 for the 3 treatment groups can be calculated (Fig. S12A). It is evident that staining intensity was low for all 3 groups at day 7, followed by increased intensity at day 14, and the highest intensity at day 21. Images from day 21 demonstrate that all treatment groups induced calcium ion deposition, with the PCL@FeWO_4_-M scaffold group exhibiting the most effective results (Fig. 6E), approximately 1.9 times higher than the PCL scaffold group and 4.1 times higher than the control group (Fig. S12B).

The differentiation of osteoblasts is a pivotal indicator within the osteogenic pathway. Osteoblastic content can be evaluated by quantitatively assessing relevant osteogenic gene expressions, such as COL1, OCN, BMP-2, and Runx2. Conventionally, quantification of the *C*_t_ values pertaining to the expression of these genes can be obtained via qPCR analysis of treated hBMSCs. Notably, the expression profiles of bone-related genes in the PCL@FeWO_4_-M group exhibited a significant up-regulation compared to both the control and bare PCL groups (Fig. 6F). This observation further verifies the enhanced capacity of PCL@FeWO_4_-M scaffolds in driving the differentiation of hBMSCs into osteoblasts. Cumulatively, the outcomes derived from osteogenic assays underscore the proficient ability of PCL@FeWO_4_-M scaffolds in facilitating hBMSC proliferation and their notable osteogenic induction capabilities in vitro. Consequently, the engineered composite scaffolds can be promising biological platforms for the remediation of bone defects.

### Evaluation of bone repair performance of PCL@FeWO4 scaffolds in vivo

To evaluate the osteogenic activity of 3D-printed PCL@FeWO_4_ scaffolds, in vivo, a cranial defect model was used in male Sprague–Dawley rats. Scaffolds were implanted into bone defects, with PCL scaffolds on the left side and PCL@FeWO_4_-M scaffolds on the right. Each scaffold measured 5 mm in diameter and 2 mm in depth [10,37]. At 4, 8, and 12 weeks after implantation, the rats were euthanized, and cranial bones were collected, fixed, sectioned, and analyzed by micro-CT and histology [38–43].

**Fig. 7.**
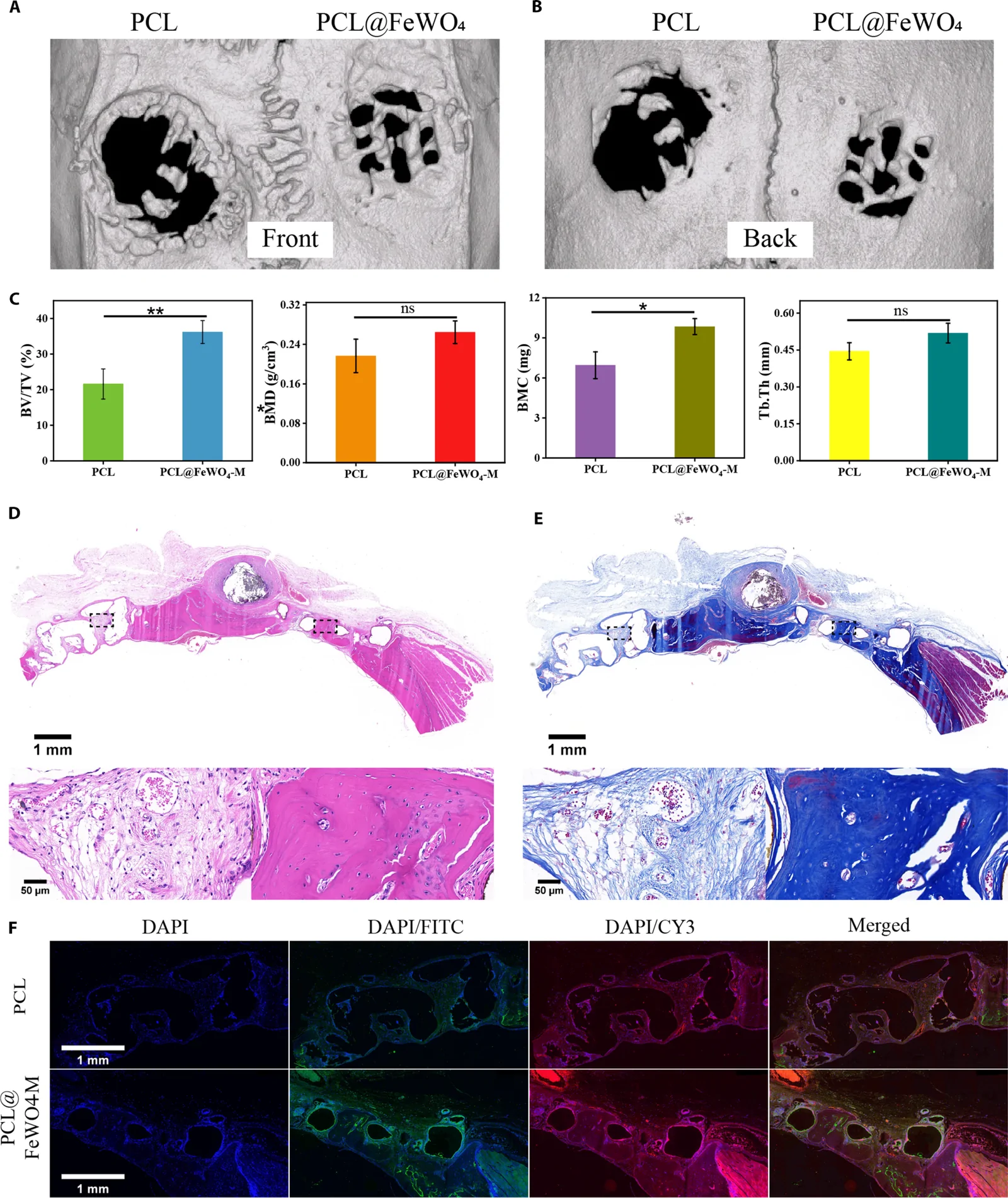
In vivo osteogenic performance of PCL@FeWO_4_-M scaffolds. (A and B) Micro-CT 3D reconstruction images of cranium harvested from experimental SD rats after 12 weeks of scaffold implantation, and images of cranial defect area with PCL and PCL@FeWO_4_-M scaffold implantation. (C) Quantitative fundamental parameters indexing the bone regeneration capability of PCL and PCL@FeWO_4_-M scaffolds based on the histomorphometric micro-CT analysis, including the relative ratio of bone volume to tissue volume (BV/TV), bone mineral density (BMD), bone mineral content (BMC), and trabecular bone thickness (Tb.Th). (D to F) Histological staining of SD rats implanted with PCL and PCL@FeWO_4_-M scaffolds after 12 weeks. Histopathological assessment of defected craniums with PCL and PCL@FeWO_4_-M scaffold implantation via (D) H&E staining and (E) Masson’s trichrome staining. (F) Immunofluorescence staining images of DAPI, DAPI/fluorescein isothiocyanate (FITC), and DAPI/CY3 from harvested craniums in different groups. Green, OPN; red, OCN. Significance levels were denoted as follows: *P < 0.05; **P < 0.01; ***P < 0.001; ns, not significant (*P* > 0.05).

Representative micro-CT images of the harvested cranial bone, along with 3D reconstructions derived from alterations in bone tissue density, corroborated the significantly enhanced reparative efficacy of PCL@FeWO_4_-M scaffolds in comparison to pure PCL scaffolds (Fig. 7A and B). Notably, the cross-linked 3D structure resembling the scaffolds remained discernible at the defect periphery, potentially indicating newly formed bone. Furthermore, observation revealed the emergence of thinly spread newly formed bone tissue traversing the interconnected defects (Fig. S13). Quantitative assessment of key parameters within the circular defect area was conducted, including BV/TV, BMD [44], BMC [45,46], and trabecular thickness (Tb.Th) [47]. As expected, PCL@FeWO_4_-M scaffolds demonstrated increased BV/TV, BMD, BMC, and Tb.Th over time, surpassing the values achieved with original PCL scaffolds (Fig. 7C). This trend suggests higher dynamics of newly formed bone tissue volume and increased average bone density of the circular defect (Fig. S14). Collectively, these findings suggested the beneficial influence of FeWO_4_ NRs in enhancing the osteogenic capability of 3D-printed scaffolds.

In addition, H&E staining and Masson’s trichrome staining were employed to assess bone tissue formation. The H&E images demonstrated substantial accumulation of mineralized bone matrix around the remaining pores of the PCL@FeWO4-M scaffolds, emphasizing the constructive contribution of their 3D porous architecture. Moreover, it was evident that PCL@FeWO_4_-M scaffolds facilitated the adherence of various proteins, migration of fibroblasts, and differentiation of hBMSCs, as depicted in Fig. 6D. Microscopic analysis of Masson’s trichrome staining further illustrated augmented collagen formation within the bone defect treated with PCL@FeWO_4_-M scaffolds (Fig. 7E). Consequently, it can be inferred that PCL@FeWO_4_-M scaffolds exhibit a propensity to expedite osteogenesis and bone tissue regeneration.

Moreover, immunohistochemistry experiments were undertaken to delve deeper into the molecular aspects. The outcomes unveiled a heightened fluorescence intensity of osteopontin (OPN) in green and osteocalcin (OCN) in red within the PCL@FeWO_4_-M group in comparison to the PCL group (Fig. 7F and Fig. S15). These findings indicate that PCL@FeWO_4_-M scaffolds promote bone regeneration by enhancing the expression of key osteogenic markers, highlighting their vital role in facilitating bone formation. Collectively, the comprehensive analysis of these findings suggests the potential of the PCL@FeWO_4_ composite scaffold as an optimal candidate for implantable bone scaffolds.

## Conclusion

In conclusion, piezoelectric FeWO_4_-integrated 3D-printing PCL scaffolds were successfully conducted by a facile impregnate method. The nanoengineered composite scaffolds feature strong piezoelectric effects that significantly facilitate the rapid generation of ROS under US irradiation. The superior piezo-sonodynamic and catalytic performance not only effectively eradicated osteosarcoma cells, exerting significant antitumor activities, but also achieved suppression and eradication of osteosarcoma xenografts with satisfactory biocompatibility. In addition, the fabricated PCL@FeWO_4_ scaffolds effectively promoted the adhesion, proliferation, and osteogenic differentiation of hBMSCs, facilitating the osteogenic process and ultimately leading to the formation of new bone tissues in a skull defect model. Overall, PCL@FeWO_4_ scaffolds may provide an inspiration to develop new type of piezoelectric nanomaterials to construct dual-functional 3D-printing scaffolds for sequential osteosarcoma therapy and bone regeneration.

## Data Availability

The data that support the findings of this study are available from the corresponding author upon reasonable request.
