# Supplementary material for: Piezoelectric Iron (II) Tungstate-Integrated Three-Dimensional-Printed Polycaprolactone Scaffolds for Ultrasound-Triggered Osteosarcoma Therapy and Ultrasound-Free Bone Repair
Source: Biomater Res. 2026 Sep 22;30:0420. doi: 10.34133/bmr.0420 (PMC13594697; doi:10.34133/bmr.0420)
Supplement: Supplementary file 1 [file bmr.0420.f1.docx]

Supplementary Materials for

Piezoelectric iron (II) Tungstate-Integrated Three-Dimensional Printed Polycaprolactone Scaffolds for Ultrasound-Triggered Osteosarcoma Therapy and Ultrasound-Free Bone Repair

Jiongming You^a,#^, Biaotong Huang^b,#^, yinsheng Wu^a^, Xinxing Sun^b^, Na Liang^b^, Hao Wang^b^, yong Wang^a,*^

*^a^ Department of Orthopedic, Wenzhou Hospital of Integrated Traditional Chinese and Western Medicine, WenZhou 325000, ZheJiang, China.*

*^b^Wenzhou Institute, Shanghai University, Wenzhou 325000, ZheJiang,China*

^#^ *These authors contributed equally: Jiongming You (yjm0401523@163.com)，Biaotong Huang (hbt021@sina.com).*

** Corresponding authors. Correspondence should be addressed to yong Wang (wy118@163.com)*

2. Supporting Figures

Fig. S1. The scheme of synthetic procedure of FeWO_4_ .

Fig. S2. The diameter and zeta potential of FeWO_4_.

Fig. S3. Schematic illustration of the construction of 3D printing PCL@FeWO_4_ scaffolds.

Fig. S4. PFM analysis of FeWO_4_ NRs.

Fig. S5. Piezoelectric constant ($\boldsymbol{d}_{\boldsymbol{33}}$) of PCL@FeWO₄ scaffolds.

Fig. S6. (a) Absorption profile of DPBF (20µM) after incubation with 1 mg/mL FeWO_4_ at time points of 0 min, 5 min, 10 min, 15 min , 20 min and 30 min. (b) Absorption profile of MB(20µM) after incubation with 1mg/mL FeWO_4_ at time points of 0 min, 10 min, 20 min, 30 min , 40 min and 50 min. Inset: digital photographs of the DPBF and MB solutions at the different time points.

Fig. S7. Time course absorbance of the chromogenic TMB when coincubated with FeWO_4_ (0.05 mg/mL) in the presence of H_2_O_2_ at varied concentration (0.02, 0.1 and 0.5M).

Fig. S8. (a) Integrated fluorescence intensity profile of ROS staining from Fig. 4d. (b) The proportion of dead cells (with red) and live cells (with green) for HOS cells from the different treatment groups.

Fig. S9. Time-dependent body weight curves of nude mice (n = 5) after different treatments (Control group,PCL@FeWO_4_-M group,US group, and PCL@FeWO_4_-M + US group).

Fig. S10. H&E staining observation of major organs (Heart, liver, spleen, lung and kidneys) from mice after treatments of Contorl, PCL@FeWO_4_, US and PCL@FeWO_4_+US (scale bar: 100 μm).

Fig. S11. Integrated fluorescence intensity profile of rhodamine phalloidin (a) and DAPI (b) from Fig. 5c.

Fig. S12. (a) Integrated density profile of Alizarin red S at 7, 14 and 21(b) days from Fig. 5e.

Fig. S13.
 Micro-CT 3D reconstruction coronal view images of cranium harvested from SD rats 4, 8 and 12 weeks after different scaffolds implantation. (Left: PCL scaffolds, Right: PCL@FeWO_4_-M scaffold)

Fig. S14. Percent bone volume (BV/TV), bone mineral density (BMD), bone mineral content (BMC) and trabecular thickness (Tb.Th) were measured using CTAn image analysis software based on microscopic CT images at 4,8,12weeks.

Fig. S15. (a-b) Quantitative analysis for the immunofluorescence staining. (n=3, **p* < 0.05, ***p* < 0.01).

3. Supporting Tables

Table S1 Primer sequences

| Gene name | Sequence (5’-3’) |
| --- | --- |
| BMP-2-F | GGGACCCGCTGTCTTCTAGT |
| BMP-2-R | TCAACTCAAATTCGCTGAGGAC |
| OCN-F | AAGCAGGAGGGCAATAAGGT |
| OCN-R | TTTGTAGGCGGTCTTCAAGC |
| COL1-F | AAGGTGTTGTGCGATGACG |
| COL1-R | TGGTCGGTGGGTGACTCTG |
| RUNX2-F | CTCTACTATGGCACTTCGTCA |
| RUNX2-R | CTTCCATCAGCGTCAACAC |
| GAPDH-F | GTGAAGGTCGGTGTGAACGG |
| GAPDH-R | TCCTGGAAGATGGTGATGGG |
